# Metatranscriptomic Analyses Unravel Dynamic Changes in the Microbial and Metabolic Transcriptional Profiles in Artisanal Austrian Hard-Cheeses During Ripening

**DOI:** 10.3389/fmicb.2022.813480

**Published:** 2022-03-01

**Authors:** Narciso Martín Quijada, Monika Dzieciol, Stephan Schmitz-Esser, Martin Wagner, Evelyne Selberherr

**Affiliations:** ^1^Department for Farm Animals and Veterinary Public Health, Unit of Food Microbiology, Institute of Food Safety, Food Technology and Veterinary Public Health, University of Veterinary Medicine Vienna, Vienna, Austria; ^2^Austrian Competence Centre for Feed and Food Quality, Safety and Innovation, FFoQSI GmbH, Tulln an der Donau, Austria; ^3^Department of Animal Science, Iowa State University, Ames, IA, United States

**Keywords:** metatranscriptomics, cheese ripening, microbial dynamics, metabolic pathways, differential gene transcription, organoleptic compounds

## Abstract

Vorarlberger Bergkäse (VB) is an artisanal Austrian washed-rind hard cheese produced from alpine cows’ raw milk without the addition of ripening cultures. Ripening time is a key factor in VB, as it strongly influences the microbial communities present in the cheeses and the organoleptic properties of the product. In this study, the microbial and metabolic transcriptional profiles in VB rinds at different ripening times were investigated. VB products before (30 days of ripening) and after (90 days of ripening) selling were selected, RNA was extracted and subjected to shotgun metatranscriptomic sequencing. The analysis revealed some of the previously described abundant bacterial taxa of *Brevibacterium*, *Corynebacterium*, *Halomonas*, *Psychrobacter*, and *Staphylococcus* to be highly active in VB rinds. Additionally, the investigation of most important metabolic pathways in cheese ripening clearly showed differences in the gene transcription profiles and the active microbiota between the two ripening points investigated. At 30 days of ripening, metabolic events related with the degradation of residual lactose, lactate, citrate, proteolysis, and lipolysis were significantly more transcribed and mainly associated with *Staphylococcus*. On the other hand, genes involved in the degradation of smaller compounds derived from previous metabolism (i.e., metabolism of free amino acids and fatty acids) were significantly more expressed in VB rinds with 90 of ripening, and mainly associated with *Brevibacterium* and *Corynebacterium*. These latter metabolic activities are responsible of the generation of compounds, such as methanethiol and 2,3-butanediol, that are very important for the flavor and aroma characteristics of cheeses. This study shows the dynamic changes in the gene transcriptional profiles associated with energy substrates metabolism and the generation of organoleptic compounds during VB ripening and uncovers bacterial taxa as key drivers of the ripening process. These taxa might be the target for future studies toward an accelerated cheese ripening and the enhancement of its organoleptic properties.

## Introduction

Cheese ripening is a complex and dynamic process in which the curd undergoes changes affecting its composition, texture, flavor, and aroma throughout ripening (McSweeney and Sousa, 2000). The metabolic activities conducted by the microbiota present in the cheese during ripening are responsible for such biochemical changes, which can be grouped into primary and secondary events. The “primary events” include the metabolism of the main compounds present in the fresh curd, such as the residual lactose, lactate, citrate, proteins (derived from proteolysis), and lipids (originating from lipolysis; McSweeney, 2004, 2017). The “secondary events” include the metabolism of those compounds that were generated after the “primary events,” such as the metabolism of free amino acids (FAA) and free fatty acids (FFA). The “secondary events” are responsible for the generation of many compounds, including alcohols, aldehydes, esters, lactones, and carboxylic acids, which have important organoleptic properties for the final products (McSweeney, 2004, 2017). The concentration of these compounds is essential for the proper organoleptic qualities of the cheese (Hassan et al., 2012).

The most abundant carbohydrates in milk are lactose, lactate, and citrate, which are mainly lost with the whey during cheese production (McSweeney and Sousa, 2000). The residual amounts of these compounds are rapidly metabolized by the starter bacteria that are added during milk coagulation and by the non-inoculated microbiota originating from the processing environment, leading to the generation of important organoleptic compounds for cheese, such as acetate, diacetyl acetoin, and 2,3-butanediol (Fox et al., 2000; Marilley and Casey, 2004; Mounier et al., 2007; McSweeney, 2017).

Proteolysis refers to the degradation of milk proteins (mainly caseins) into small peptides and, ultimately, into FAA (Dugat-Bony et al., 2015). Although peptides and FAA are known to affect cheese flavor and aroma, the greatest influence comes from the broad spectrum of compounds generated by FAA catabolism, which are usually used as energy substrates after exhaustion of lactate (also called “carbohydrate starvation”; Mounier et al., 2007; Ardö et al., 2017). It is worth noting the importance of sulfur-containing amino acids (mainly methionine, as it is more abundant than cysteine in milk), because their metabolism produces a broad range of low molecular weight volatile sulfur compounds (VSC), such as methanethiol, whose relevance for the final products’ flavor and aroma has been stated before (Weimer et al., 1999; McSweeney and Sousa, 2000; Ganesan and Weimer, 2017).

Cow milk contains an average of 35 g/l of lipids (mainly triglycerides) that result in the generation of FFA after lipolysis (Monnet et al., 2015). FFA can also be produced after FAA metabolism, and are important precursors of catabolic reactions producing volatile compounds with important organoleptic properties (Sourabié et al., 2012; Thierry et al., 2017).

Despite of the availability of important biochemical knowledge in the ripening process, the study of the gene expression patterns of cheese rind microbial communities is still limited. These analyses provide useful insights into the cheese microbial activities, paving the way to optimizing the manufacturing process, and ensure the optimal quality and safety of the final products. Such information is strongly appreciated by cheesemakers, who are frequently faced with challenges of inconsistent unstable ripening activity, the growth of undesirable microorganisms, cheese defects and the increasing demands from the market.

Austrian Vorarlberger Bergkäse (VB) is an artisanal raw milk brine-washed hard cheese manufactured in the western part of Austria (Vorarlberg) that has a protected designation of origin according to the Council Regulation (EEC) of the European Union No. 2081/92. VB is produced without the addition of external ripening cultures and the cheese wheels are brined at different concentrations and frequencies depending on the product during the ripening, which can last from three to 18 months. The ripening time is a key factor for the organoleptic properties of VB that vary widely between the different times and have a significant impact on the consumer market.

Recently, VB and its associated microbiota have been studied in detail by our group using different culture-dependent and independent approaches. Certain bacterial taxa, such as *Brevibacterium*, *Corynebacterium*, *Halomonas*, and *Staphylococcus* species, have been found to be highly abundant on VB rinds throughout ripening and in the processing environment, according to 16S rRNA gene cloning and amplicon sequencing (Schornsteiner et al., 2014; Quijada et al., 2018). Additionally, the genomes of *Advenella*, *Brevibacterium, Psychrobacter*, and *Psychroflexus* strains isolated from the VB rinds were investigated by using whole genome sequencing and a putative metabolic pathway for histamine degradation was found in different *Brevibacterium* strains from VB (Schmitz-Esser et al., 2018; Anast et al., 2019). More recently, the bacterial and fungal diversity of VB rinds was evaluated by high-throughput gene-targeted sequencing at different times throughout VB ripening, unraveling microbial dynamics and potential correlations of different bacteria and fungi during ripening (Quijada et al., 2020b). Such microbial correlations may be critical for the unique organoleptic properties of the VB products that are sold at different time points during ripening.

In this study, shotgun metatranscriptomic analyses were conducted with the aim to investigate the metabolic events that are highly transcribed in VB ripening and to gain insight into the potential molecular mechanisms and biotic relationships occurring between the fresh cheese and when it is sold. Rind samples were taken from VB wheels after 30 days and 90 days of ripening, and nucleic acids were extracted and subjected to DNA- and RNA-based shotgun sequencing.

## Materials and Methods

### Cheese Production and Sampling

The VB cheese rind samples included in this study were taken from a long-established facility in the region of Vorarlberg (Austria). For the cheese production, cow’s raw milk is used and only traditional cheese ripening techniques are applied. Starter cultures (Federal Institute for Alpine Dairying, Rotholz, Austria) included *Streptococcus thermophilus, Lactococcus delbrueckii* spp. *lactis*, and *Lacticaseibacillus casei.* Dry salting of cheese was applied during day 0–6 (45–50 g NaCl per side, each side three times). The ripening temperature was 13°C and humidity was 93–94%. Cheeses were treated with brine 2–3 times a week with a brine concentration of 10%. Samples were taken from six different cheeses after 30 (from three different cheeses of the same production batch) and 90 (from three different cheeses of the same production batch) days of ripening. VB-cheese rinds were harvested by scraping the cheese rind with sterile scalpels and stored immediately in RNA*later*™ (Invitrogen, Thermo Fisher Scientific, Austria) to stabilize mRNA. Samples were stored at 4°C during transport to the laboratory and subsequently frozen at −80°C.

### Nucleic Acid Extraction and Sequencing

RNA from 250 mg cheese rinds was extracted from RNAlater-preserved samples using the innuSPEED Bacteria/Fungi RNA kit according to the manufacturer’s instructions (Analytik Jena AG, Germany). Briefly, samples were mixed after thawing, and RNAlater solution was removed prior to extraction by a centrifugation step for 10 min at 5,000 × g. The RNA pellet was rehydrated in 20 μl nuclease-free water and stored at −80°C until use. Subsequently, DNA was digested by a treatment of the RNA samples with Turbo DNA-free™ kit (Ambion, United States) according to the manufacturer’s instructions. Purified RNA was quantified with Qubit^®^ RNA BR assay kits of the Qubit^®^ fluorometer 2.0 (Invitrogen, Vienna, Austria). Additionally, to confirm that the samples were free of DNA contaminants, 16S rRNA gene PCRs with the primers 27F (5′- AGAGTTTGATCMTGGCTCAG-3′) and 1492R (5′-GGYTACCTTGTTACGACTT-3′) as well as with 515F (5′-GTGCCAGCMGCCGCGGTAA-3′) and 806R (5′-GGACTACVSGGGTATCTAAT-3′) were performed. The qualitative and quantitative analysis of RNA samples (e.g., RIN^e^, RNA integrity number equivalent) was performed using the Agilent 4200 TapeStation system (Agilent Technologies, United States) and the TapeStation software revision A.02.02 (SR1). Samples were then subjected to further library preparation and shotgun metatranscriptomic sequencing.

In order to have a metagenomic template from the VB rinds samples that could work as a close reference for the metatranscriptomic dataset of this particular product, two cheese rind samples (one for 30 days and for 90 days of ripening) were chosen for DNA extraction and DNA-based metagenome shotgun sequencing. Total DNA from cheese rind samples was extracted by using the DNeasy PowerSoil Pro kit (Qiagen) according to the manufacturer’s instructions. DNA concentrations were determined with a Qubit^®^ 2.0 Fluorometer (Thermo Fisher Scientific, Vienna, Austria) and the presence of bacterial DNA was addressed by gel electrophoresis of 16S rRNA gene amplicon PCR products (primers 27F/1492R).

Library preparation, rRNA depletion (NEBNext^®^ rRNA Depletion kit), transcription into cDNA, library quality control, and shotgun sequencing were performed at the Vienna BioCenter Core Facilities GmbH (Vienna, Austria) using an Illumina HiSeqV4 platform; which yielded a mean of 211.6 M metagenomic (125 bp paired end reads, PE) and 83.8 M metatranscriptomic (50 bp single end reads, SE) reads per sample.

### Bioinformatic Analysis

#### Sequencing Data Processing

The sequencing data provided as BAM files was converted into FASTQ files by using BedTools v2.29.2 and Samtools 1.7 (Li et al., 2009). Quality control of the sequencing reads was assessed by using FASTQC v0.11.9.^1^ Residual sequencing barcodes and adapters were removed, and quality filtering of the reads were performed by using Trimmomatic v0.39 (Bolger et al., 2014). The presence of rRNA reads in the metatranscriptomic dataset was screened and removed by using SortMeRNA v4.3.1 (Kopylova et al., 2012). Despite the rRNA depletion prior to sequencing, the rRNA reads content was significant, and the *in silico* rRNA depletion removed approximately 65% of the reads from the metatranscriptomic dataset.

#### Metagenomic and Metatranscriptomic Analysis

The metagenomic and metatranscriptomic analysis was performed by using the SqueezeMeta pipeline v1.1.1 (Tamames and Puente-Sánchez, 2019) with some modifications as follows: co-assembly of the metagenomic samples was performed by using SPAdes v3.11.1 (Bankevich et al., 2012), contigs below 200 bp were removed by using Prinseq (Schmieder and Edwards, 2011), and assembly statistics were conducted by QUAST (Gurevich et al., 2013). The metatranscriptomic reads were excluded for the assembly process by properly modifying the required input metadata file for SqueezeMeta. The assembly yielded a total length over 195 Mbp divided in 141,192 contigs (*N*_50_ = 5,257 bp and average GC content of 59.02%). Open reading frames were predicted by using Prodigal v2.6.3 (Hyatt et al., 2010), identifying a total of 287,698 coding sequences (CDS). tRNA and tmRNA prediction was performed by using Aragorn v1.2.38 (Laslett and Canback, 2004). Similarity searches of the predicted proteins against the GenBank (Clark et al., 2016) and Kyoto Encyclopedia of Genes and Genomes (KEGG) (Kanehisa and Goto, 2000) databases was performed by using Diamond v0.9.22.123 (Buchfink et al., 2014). Overall, 244,504 (85.0% of all proteins) and 137,328 (47.7%) proteins were annotated against the GenBank and KEGG databases, respectively. Mapping of the metagenomic reads against the co-assembled contigs was performed by using Bowtie2 v2.3.4.1 (Langmead and Salzberg, 2012) and resulted in >99% of the reads mapping, showing that the vast majority of the metagenomic reads were included in the co-assembly.

The metatranscriptomic SE reads were mapped against the co-assembly by using Bowtie2 and read counts were normalized by using the *sqm-count* algorithm included in SqueezeMeta. Transcript per million (TPM) values were calculated for the KEGG orthologues (KO) features. In order to describe the gene transcription profiles for each gene, percentiles (P) based on the total TPM values were calculated and transcription levels were classified as “very low” (when the TPM of the gene was <*P*_25_), “low” (>*P*_25_, <*P*_50_), “medium” (>*P*_50_, <*P*_75_), “high” (>*P*_75_, <*P*_90_) and “very high” (>*P*_90_). The mapping ratio was over 90% for all samples (except for sample S6), suggesting that the samples selected for DNA sequencing and further co-assembly represented an appropriate and close reference for the VB rind metatranscriptomic analysis. The mapping ratio for S6 was below the others (41%). This sample also yielded the lowest number of reads passing the quality control and the *in silico* rRNA removal. Further attempts to classify the reads from S6 that did not map against the co-assembly were performed *via* an individual assembly with SPAdes and by using the *SQM_reads.pl* tool included in the SqueezeMeta pipeline, but none revealed any further information.

Differential gene expression (DGE) analysis was performed in R environment v3.6.1 (R Core Team, 2019) by using DESeq2 v1.26.0 (Love et al., 2014). Genes with less than 10 mapped reads overall were discarded for DGE analysis. Raw values of *p* were adjusted for multiple testing using the Benjamini and Hochberg (1995), which assesses the False Discovery Rate. Gene transcripts with an adjusted *p* < 0.05 were considered to be differentially transcribed between the two ripening times investigated. Complete description of the genes identified in the study, together with their corresponding TPM values and DGE significances are shown in Supplementary Material 1.

#### Visualization of the Data

Visualization of the data was performed under R environment v.3.6.3. The SQMTools package v0.6.0 (Puente-Sánchez et al., 2020) was used for managing and inspecting the output data resulting from the SqueezeMeta pipeline, as well as sub-setting the count files for particular taxa and function of interest (by using the *subsetTax* and *subsetFun* options). The R packages ggplot2 v3.3.3 (Wickham, 2011), reshape v1.4.4 (Wickham, 2007) and ggpubr v0.4.0 (Kassambara, 2020) were used for the visualization of the data and the generation of figures. Further customization of the vector images, when needed, was performed with Inkscape v.0.91 [Inkscape Project (2020). *Inkscape*].^2^

## Results

### Overview of the Shotgun Sequencing Data

**Figure 1:**
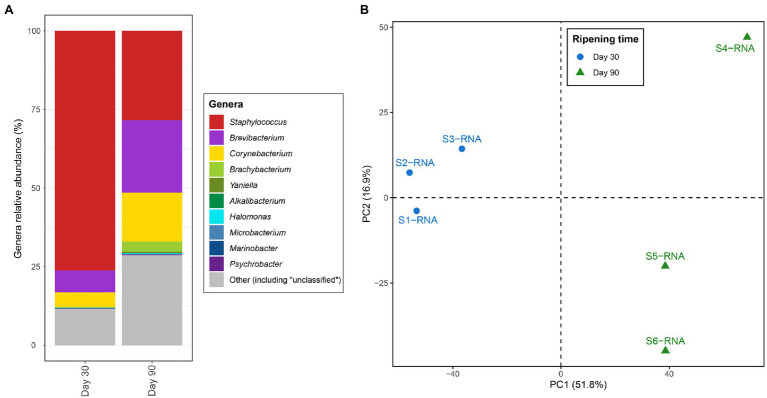
**(A)** Percent of transcript aligned to the coding sequences assigned to the different genera identified in VB rinds. The “Other” section includes other genera as well as genomic regions where the genus-level resolution was not achieved. **(B)** Principal component analysis (PCA) based on the transcription values [calculated as transcripts per million (TPM)] assigned to the different Kyoto Encyclopedia of Genes and Genomes (KEGG) orthologues and samples.

After quality control, a mean of 201 M metagenomic reads per ripening time were obtained and co-assembled to achieve a close reference for the metatranscriptomic analysis. The metagenomic assembly yielded 141,192 contigs (*N*_50_ = 5,257 bp and GC content = 59.02%) with an overall length of 195 Mbp. A total of 287,698 CDS were identified, where 244,504 CDS (85.0% of all CDS) got a taxonomic annotation against the GenBank database and 137,328 CDS (47.7%) got a functional annotation against the KEGG database (and grouped into 6,076, KO). Overall, an average of 30 M transcripts per sample remained after the quality filtering and the removal of residual rRNA. To estimate the taxonomy of the most active microorganisms, the percentage of the normalized transcripts that were assigned to CDS with taxonomic annotations was calculated and shown in Figure 1A. Overall, 99.7% of the metatranscriptomic reads were assigned as Bacteria (52.6% of them were assigned as *Actinobacteria* and 46.6% as *Firmicutes*) and 0.2% as Fungi (99.9% of them were assigned as *Ascomycota*).

Figure 1A shows differences in the most active microbiota in VB rinds at the two ripening times investigated. *Staphylococcus* was the most active bacteria in VB rinds at 30 days of ripening, with large numbers of the transcripts mapping against CDS assigned to this genus (almost 75% of the reads). At 90 days of ripening, the number of reads assigned to *Staphylococcus* decreased in comparison to 30 days of ripening; however, still representing 28.4% of the transcripts at this ripening time. The transcription profiles of *Brevibacterium* and *Corynebacterium* increased significantly through ripening, as the percentage of normalized reads assigned to these genera increased from 7.0 to 23.1% from day 30 to 90 in VB rinds for *Brevibacterium* and from 4.7 to 15.5% for *Corynebacterium*.

From the 6,076 KO identified in the VB rind metagenomic dataset, 4,846 (79.8%) had at least one transcript aligning to them. The entire list and description of the KO identified in the study, with the average TPM and log_2_ fold change values per condition is shown in Supplementary Material 1. The number of transcripts mapping to each KO and to each sample was normalized to TPM, and the resulting distance matrix was analyzed and visualized as a principal components analysis (PCA) in Figure 1B. The results clearly show that the metabolic profiles varied in VB rinds at 30 (blue) and 90 (green) days of ripening, as the samples clustered together according to the same ripening time and apart from the other group.

The following sections of the manuscript will describe some of the key metabolic pathways for cheese ripening and how these pathways were represented in VB rinds in the two ripening times investigated, as well as the most active microorganisms.

### Metabolism of Residual Lactose, Lactate, and Citrate

**Figure 2:**
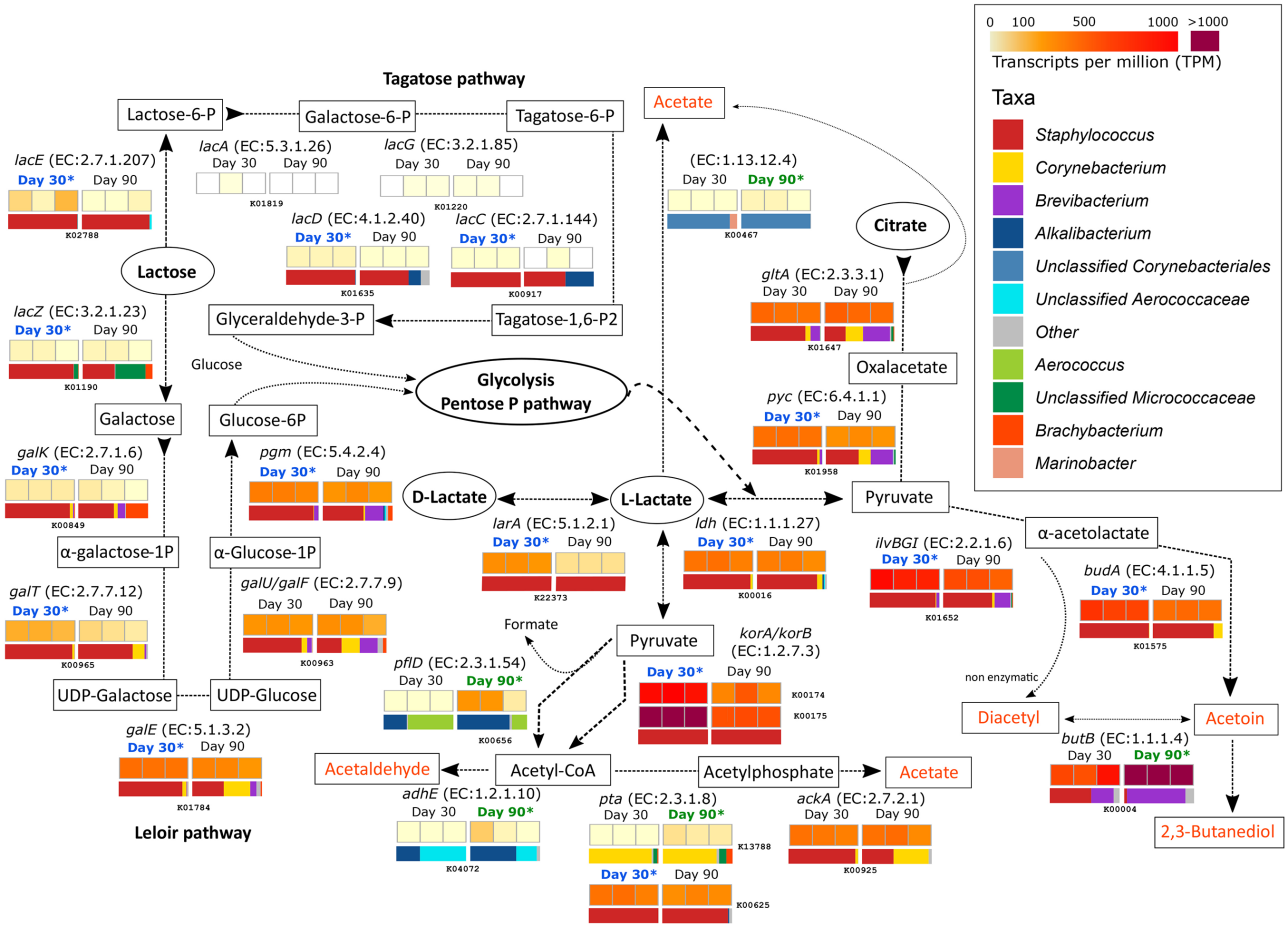
Metabolism of residual lactose, lactate and citrate. The different compounds that are generated in each metabolic pathway are showed and those harboring important organoleptic properties for cheese are colored in orange. The different metabolic pathways can be followed according to the corresponding arrows. Between every pair of compounds, the enzyme that catalyzes each reaction is listed (gene name and EC number). Below each enzyme are six squares showing the TPM values for each of the six different samples. The TPM color scale appears in the legend in the upper-right part of the figure. Above the TPM values, the ripening time is highlighted if significant differences occur in the transcription of that gene in that ripening time (*p* < 0.05). The horizontal bar-plot below the TPM values correspond to the percentage of transcripts assigned to the different taxa.

The main metabolic events involving the catabolism of the residual lactose, lactate, and citrate, together with the gene transcription profiles and the microbiota involved, were investigated and summarized in Figure 2. The large representation of *Staphylococcus, Brevibacterium*, and *Corynebacterium* can be observed in most of the metabolic pathways described in this section.

The degradation of lactose by both the tagatose and Leloir pathways was significantly more active in samples from day 30 and was mainly driven by *Staphylococcus*. The Leloir pathway, which includes the degradation of the galactose moiety of lactose after *lacZ* (K01190, EC:3.2.1.23) action, showed higher transcription values than the tagatose pathway. The products of lactose degradation, glucose-6-P and glyceraldehyde-3-P then undergo glycolysis and/or the pentose phosphate pathway, which releases pyruvate and L-lactate as intermediate products.

Lactate can be formed from pyruvate after glycolysis by the action of lactate dehydrogenase (LDH, EC:1.1.1.27), encoded by the *ldh* gene (K00016). This gene was highly transcribed overall and significantly more transcribed in samples from 30 days of ripening and mainly originated from *Staphylococcus* (2-fold increase at day 30). The same enzyme can convert pyruvate from L-lactate. L-lactate can be converted to its isomer, D-lactate, by the lactate racemase *larA* (K22373, EC:5.1.2.1) in an equilibrium that is important for the texture of cheese. This gene was significantly more transcribed at 30 days of ripening in *Staphylococcus*. The metabolism of lactate involves its conversion into pyruvate (by LDH) that can be further metabolized to acetyl-CoA by following different pathways (see also “Metabolism of Other Amino and Fatty Acids” section below). Two of the most active genes in this conversion were *korA* (K00174) and *korB* (K00175), which encode for 2-oxoglutarate synthase (EC:1.2.7.3). These genes were significantly associated with *Staphylococcus* in VB rinds at 30 days of ripening (6.5- and 5.3-fold increases for *korA* and *korB*, respectively). Acetyl-CoA can be involved in multiple and important metabolic reactions, some of which can generate organoleptic compounds important for the cheese (organoleptic compounds are highlighted in orange in Figure 2). Acetyl-CoA can be metabolized to acetaldehyde by the action of the acetaldehyde dehydrogenase (EC:1.2.1.10) coded by *adhE* (K04072). *adhE* was significantly more active in samples from day 90 and was associated with unclassified *Corynebacteriales* and *Aerococcaceae*. Acetyl-CoA can also be converted to acetate, although not significant differences were identified between the two time points.

As shown in Figure 2, the genes involved in the metabolism of citrate were highly transcribed in both ripening conditions. The first steps in this metabolisms were significantly more transcribed at day 30 by *Staphylococcus*, while the last were significantly more transcribed in day 90 by *Brevibacterium*. Citrate is metabolized to oxaloacetate, pyruvate, α-acetolactate, and finally acetoin by a series of reaction involving *pyc* (K01958)*, ilvB/ilvG/ilvI* (K01652), and *budA* (K01575) genes. The enzymes encoded by these genes, mainly assigned to *Staphylococcus* and significantly more transcribed at day 30 (2.8-, 2.6-, and 4-fold increments for *pyc, ilvB/ilvG/ilvI*, and *budA,* respectively), but they were highly active at both time points. Acetoin can be converted to diacetyl and 2,3-butanediol by the diacetyl reductase (EC:1.1.1.103) and butanediol dehydrogenase (EC:1.1.1.4), both encoded by *butB* (K00004). These compounds are very important for cheese flavor and aroma. *butB* was among the 35 most transcribed genes overall and was most significantly transcribed in *Brevibacterium* in VB rinds after 90 days of ripening (3.2-fold increase).

### Proteolysis and Lipolysis

The degradation of the proteins and lipids present in the curd are pivotal for the release of smaller polymers that can in turn be the source of subsequent reactions yielding organoleptic compounds. Such reactions are conducted by proteases, peptidases, lipases, and esterases. The genes encoding for some of these enzymes were among the most transcribed genes overall.

The most transcribed proteolytic genes overall *pepA* (K01261, coding for glutamyl aminopeptidase, EC:3.4.11.7), *pepF* (K08602, oligoendopeptidase F, EC:3.4.24.-), *pepQ* (K01271, Xaa-Pro dipeptidase, EC:3.4.13.9), and *pepT* (K01258, tripeptide aminopeptidase, EC:3.4.11.4) were significantly more transcribed at day 30 and mainly associated with *Staphylococcus* (2.9-, 3.0-, 1.7- and 3.3-fold increases for *pepA, pepF, pepQ*, and *pepT*, respectively; Supplementary Material 1). Other peptidases, such as *pepI* (K01259, prolyl aminopeptidase, EC:3.4.11.5)*, pepN* (K01256, membrane alanyl aminopeptidase, EC:3.4.11.2), and *pepO* (K07386, putative endopeptidase, EC:3.4.24.-) were significantly more transcribed in samples at 90 days of ripening (>2-fold change compared to day 30) and associated with *Brevibacterium* and *Corynebacterium*, although their overall transcription was significantly lower than the genes described before.

The transcription of the genes coding for the most important lipases and esterases undergoing the first steps of lipolysis were investigated, revealing *lip* (K01046, triacylglycerol lipase, EC:3.1.1.3), *pldB* (K01048, lysophospholipase, EC:3.1.1.5), and *yvaK* (K03928, carboxylesterase, EC:3.1.1.1) to be very highly transcribed in VB rinds and significantly associated with *Staphylococcus* at day 30 (2.8-, 4.6-, and 2.6-fold increases, respectively; Supplementary Material 1).

### Metabolism of Amino Acids and Fatty Acids

#### Metabolism of Sulfur-Containing Amino Acids

**Figure 3:**
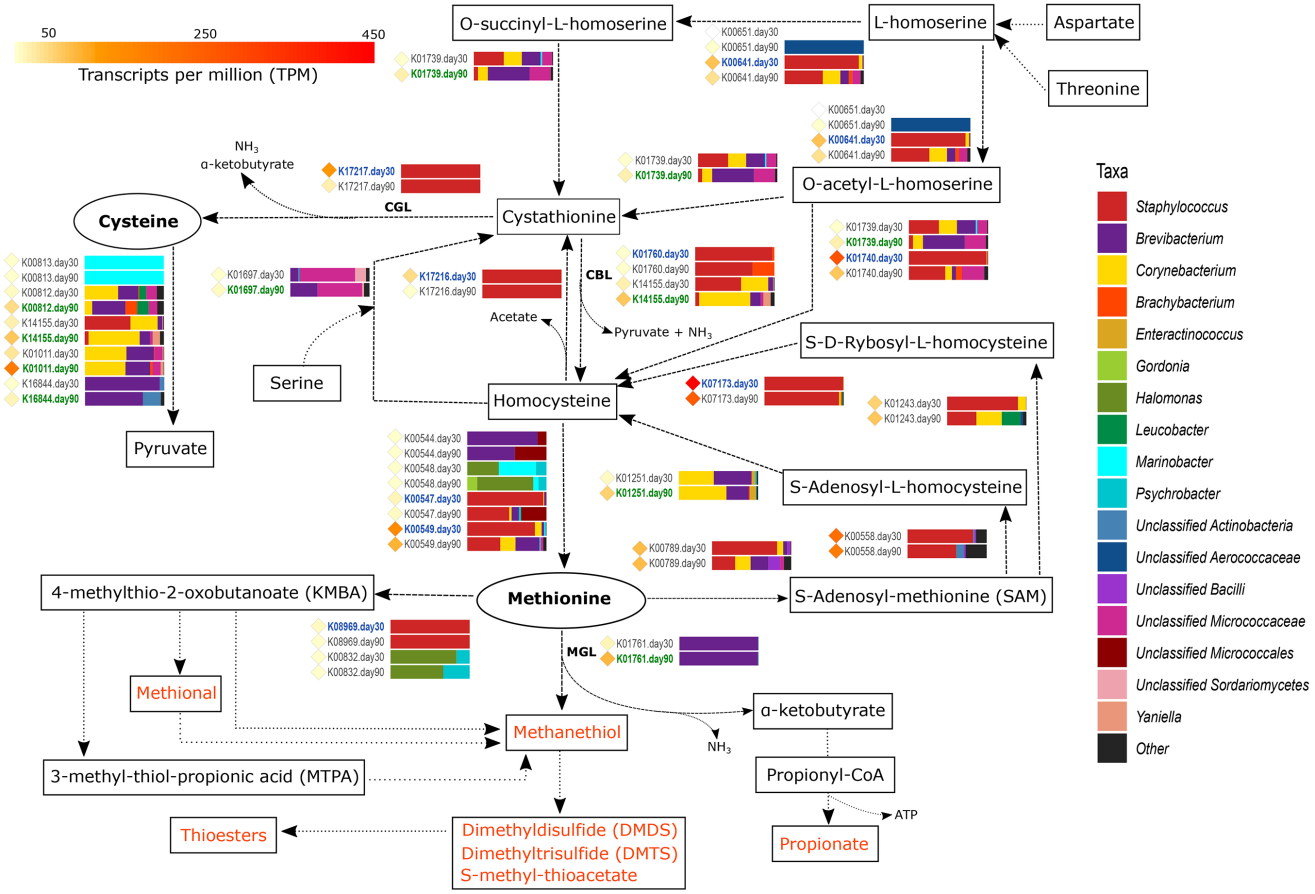
Metabolism of sulfur-containing amino acids. The different compounds that are generated in each metabolic pathway are showed and those harboring important organoleptic properties for cheese are colored in orange. The different metabolic pathways can be followed according to the corresponding arrows. Between every pair of compounds, the genes involved in such reactions are shown with their KEGG orthologue (KO) name. TPM values of each gene are shown as colored diamonds next to their corresponding KO name, which is colored in blue or green if significant transcription was identified for such KO day 30 or 90, respectively (*p* < 0.05). Next to the KO names, the horizontal bar-plots represent the percentage of transcripts for that KO and ripening time that was associated with the different taxa.

Volatile sulfur compounds are very important organoleptic compounds, as they contribute with garlic and cooked cauliflower and cabbage flavor and aroma characteristics to cheeses. They are derived mainly from the degradation of the two sulfur-containing amino acids methionine and cysteine. The main reactions and genes involved in the metabolism of these two amino acids toward the generation of important organoleptic compounds, together with the most active microbiota, are described in this section and summarized in Figure 3.

From all VSCs associated with cheese, methanethiol is the one that receives most attention. Methanethiol can be formed from methionine in a single-step reaction conducted by methionine-γ-lyase (MGL, EC:4.4.1.11), with the concomitant generation of α-ketobutyrate and ammonia. The transcription of the gene coding for methionine-γ-lyase (K01761) was significantly higher in VB rinds at 90 days of ripening (4.4-fold increase) and was fully associated with *Brevibacterium*. Methanethiol can be further chemically or enzymatically oxidized to other sulfur-bearing compounds with important organoleptic properties, such as dimethyl disulfide (DMDS), dimethyl trisulfide (DMTS), or react with fatty acids to generate thioesters. The α-ketobutyrate generated concomitantly with methanethiol is essential for the energy production of the cell *via* production of propionate (see the “Metabolism of Other Amino Acids and Fatty Acids” section). Methanethiol can be also produced from methionine by the action of cystathionine-β-lyase (cysteine-*S*-conjugate β-lyase, CBL, EC:4.4.1.13) and cystathionine-γ-lyase (CGL, EC:4.4.1.1), although this non-specific conversion is less efficient than that conducted by MGL. The main function of CBL and CGL enzymes is the elimination of cystathionine, either by converting it to homocysteine, pyruvate and ammonia or to cysteine, α-ketobutyrate, and ammonia, respectively. The genes encoding for CBL, *metC* (K01760) and *patB* (K14155), and for CGL, *mccB* gene (K17217), showed a medium transcription overall. *patB* showed a higher transcription than *metC* and was significantly more transcribed in *Corynebacterium, Brevibacterium*, and *Yaniella* in VB rinds with 90 days of ripening (2.2-fold change). On the other hand, the *mccB* gene was significantly more transcribed in *Staphylococcus* at day 30 (9.9-fold increase).

The two sulfur-containing FAA, cysteine and methionine, are connected through homocysteine and cystathionine by a series of reactions that are shown in Figure 3 and explained in detail in Supplementary Material 2. Additionally, other FAA, such as aspartate, threonine and serine can be included in the cycle by the action of different enzymes (Supplementary Material 2). The metabolism of cysteine can generate pyruvate that is involved in important metabolic reactions including the generation of important organoleptic compounds (as described below). *sseA* (K01011) codes for this reaction, was highly transcribed overall and significantly more transcribed in VB rinds from day 90, associated mainly with *Brevibacterium* and *Corynebacterium*.

#### Metabolism of Aromatic Amino Acids

**Figure 4:**
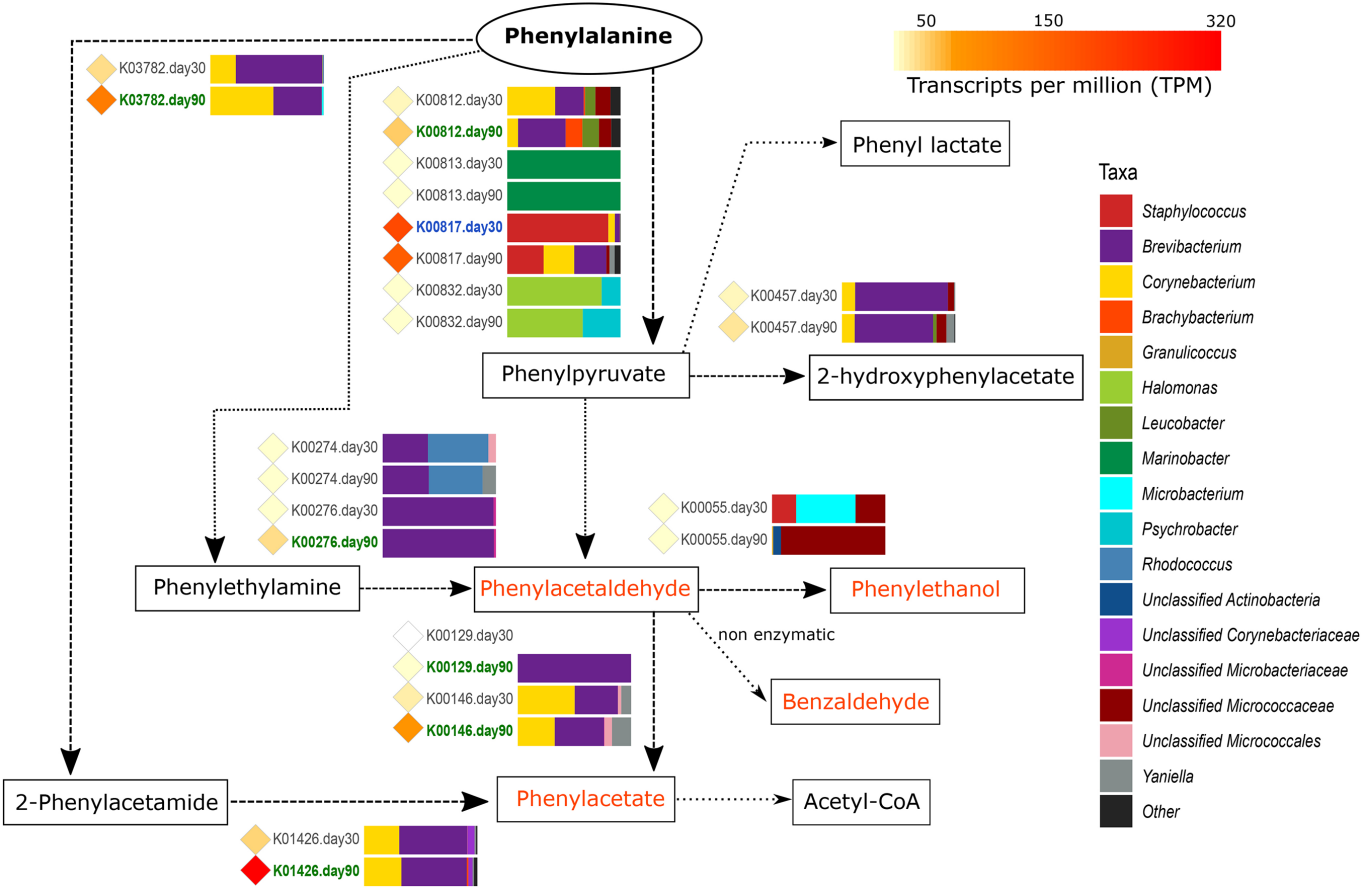
Metabolism of phenylalanine. The different compounds that are generated in each metabolic pathway are showed and those harboring important organoleptic properties for cheese are colored in orange. The different metabolic pathways can be followed according to the corresponding arrows. Between every pair of compounds, the genes involved in such reactions are shown with their KO name. TPM values of each gene are shown as colored diamonds next to their corresponding KO name, which is colored in blue or green if significant transcription was identified for such KO day 30 or 90, respectively (*p* < 0.05). Next to the KO names, the horizontal bar-plots represent the percentage of transcripts for that KO and ripening time that was associated with the different taxa.

The metabolism of aromatic amino acids (phenylalanine, tryptophan, and tyrosine) is another important aspect in cheese ripening, as it leads to a variety of products conferring rosy, floral, and fruity aromas and flavors to the cheeses during aging. These amino acids undergo similar catabolic degradation: the FAA is transaminated to produce an aromatic pyruvate, which is further reduced to an aromatic acid, an aldehyde, and an alcohol. Among the three aromatic FAA, the genes involved in the catabolism of Phe were the most transcribed overall. The main steps of Phe catabolism and the microbiota involved in each reaction are summarized in Figure 4.

The catabolism of phenylalanine to phenylpyruvate begins with the action of different transaminases (EC: 2.6.1) that can also be involved in other transamination reactions. From the genes coding for transaminases involved in this reaction, *hisC* (K00817, histidinol-phosphate aminotransferase, EC:2.6.1.9) was found to be highly transcribed overall in VB rinds and significantly associated with *Staphylococcus* at day 30 (1.9-fold increase).

As it can be seen in Figure 4, once the phenylpyruvate is formed, it can be the source for multiple reactions yielding organoleptic compounds that were mainly conducted by *Brevibacterium* and *Corynebacterium* at 90 days of ripening. Phenylpyruvate can be reduced to phenylacetaldehyde, a compound with important organoleptic properties which can be further converted to other organoleptic compounds such as phenylethanol, benzaldehyde, and phenylacetate. Phenylacetaldehyde can be formed by the action of deaminases (oxidoreductases), such as the primary-amine oxidase (EC:1.4.3.21) coded by *tynA* (K00276). *tynA* was significantly more transcribed in *Brevibacterium* from VB rinds at 90 days of ripening (11.6-fold increase). Phenylacetate can be formed from phenylacetaldehyde (by the action of dehydrogenases) or from phenylalanine (with 2-phenylacetamide as intermediate product). As shown in Figure 4, the main genes involved were significantly more transcribed in *Brevibacterium* and *Corynebacterium* at 90 days of ripening: *feaB* (K00146, phenylacetaldehyde dehydrogenase, EC:1.2.1.39), *katG* (K03782, catalase peroxidase, EC:1.11.1.21), and *amiE* (K01426, amidase EC:3.5.1.4).

#### Metabolism of Other Amino Acids and Fatty Acids

**Figure 5:**
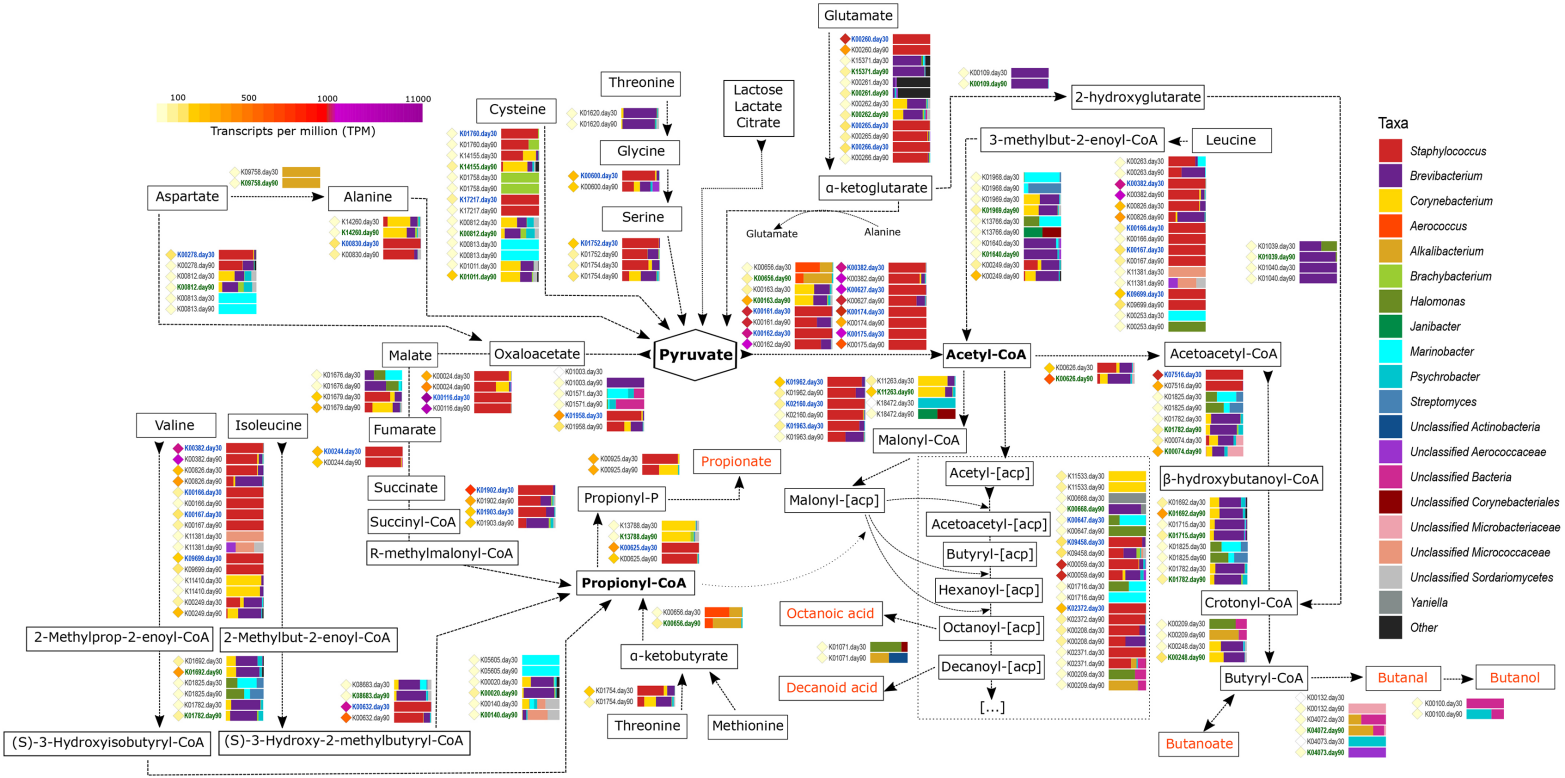
Metabolism of other amino acids and fatty acids. The different compounds that are generated in each metabolic pathway are showed and those harboring important organoleptic properties for cheese are colored in orange. The different metabolic pathways can be followed according to the corresponding arrows. Between every pair of compounds, the genes involved in such reactions are shown with their KO name. TPM values of each gene are shown as colored diamonds next to their corresponding KO name, which is colored in blue or green if significant transcription was identified for such KO day 30 or 90, respectively (*p* < 0.05). Next to the KO names, the horizontal bar-plots represent the percentage of transcripts for that KO and ripening time that was associated with the different taxa.

The metabolism of FAA and FFA is strongly connected with the generation of ATP and redox compounds such as NADH and NADPH. The metabolic degradation of these compounds is more relevant in cheese during aging, after the so called “carbohydrate starvation,” and release compounds with important organoleptic properties for the cheese. A summary of some of the metabolic events occurring in VB rinds encompassing the metabolism of FAA and FFA can be observed in Figure 5 and Supplementary Material 3. The complete description of the genes described is summarized in Supplementary Material 1.

The catabolism of branched-chain amino acids (leucine, isoleucine, and valine) involves a series of reactions that generate different branched-chain fatty acids with important organoleptic properties. The first steps in the degradation of these amino acids are similar between them and involves the action of amino transferases, dehydrogenases, and acyl transferases that generate acyl-enoyl-CoA compounds as intermediate products. Most of the genes coding for the enzymes involved in these conversions were significantly more transcribed at day 30 of ripening in *Staphylococcus* and were among the most transcribed genes in the entire study overall (Figure 5). That was the case of *pdhD* (K00382, dihydrolipoamide dehydrogenase, EC:1.8.1.4), *bkdA2* (K00167, 2-oxoisovalerate dehydrogenase E1 component beta subunit, EC:1.2.4.4), and *bkdB* [K09699, 2-oxoisovalerate dehydrogenase E2 component (dihydrolipoyl transacylase), EC:2.3.1.168]. The resultant intermediate acyl-enoyl-CoA compounds could be converted to their corresponding branched chain FFAs by the action of phosphatases and acyl kinases, with the concomitant generation of ATP. The next steps in the metabolism of these FAA differs, as leucine is converted into acetyl-CoA and valine and isoleucine into propionyl-CoA (Figure 5). Strikingly, most of the genes involved in the latest reactions of these FAA were significantly more transcribed at day 90 and mainly associated with *Brevibacterium* and *Corynebacterium*.

Propionyl-CoA can also be generated from threonine and methionine after their conversion into α-ketobutyrate (with the concomitant generation of methanethiol in the case of methionine, as described before; Figure 5). The conversion of α-ketobutyrate to propionyl-CoA is controlled by *pflD* (K00656, formate *C*-acetyltransferase, EC:2.3.1.54), which was significantly more transcribed at day 90 in *Brachybacterium* and *Alkalibacterium*. Additionally, propionyl-CoA can generated from pyruvate after the tricarboxylic acids (TCA) cycle *via* R-methylmalonyl-CoA (Figure 5). Propionyl-CoA can be further metabolized to generate propionate that confers organoleptic properties for the cheese, with the concomitant generation of ATP.

Pyruvate has a key role in cellular metabolism and can be generated from lactose, lactate, and citrate (as described in the previous sections), as well as from different FAA, including alanine, cysteine, glutamate, and serine (Figure 5 and Supplementary Material 3). Alanine and glutamate are abundant FAA in milk and their metabolism increases after the “carbohydrate starvation.” Alanine metabolism involves transamination or deamination reactions to yield pyruvate. Several genes are involved in this step, such as *AGXT* (K00830, alanine-glyoxylate transaminase, EC:2.6.1.44), which was highly transcribed and significantly more transcribed at day 30 in *Staphylococcus*. Glutamate can be degraded to α-ketoglutarate with the generation of NAD(P)H and ammonia by the action of dehydrogenases and transaminases. *gudB* (K00260, glutamate dehydrogenase, EC:1.4.1.2) was very highly transcribed overall and significantly associated with *Staphylococcus* at 30 days of ripening (Figure 5). The generated α-ketoglutarate can enter the TCA cycle or be used as a substrate for transamination reactions and favors the catabolism of other FAAs, as they work as amino group acceptors.

As described before, FFA may originate from the degradation of milk fats by the action of lipases and esterases. Additionally, they can also be formed after the breakdown of FAA or synthesized *ex-novo* from the pyruvate obtained after FAA catabolism (Figure 5). Pyruvate can be converted to acetyl-CoA by different reactions (as also described in the “Metabolism of Residual Lactose, Lactate And Citrate” section, Figure 2). Acetyl-CoA receives the acyl carrier protein (acp) from Malonyl-[acp]. Malyonyl-[acp] is also produced from acetyl-CoA involving a series of genes that were mainly associated with *Staphylococcus, Brevibacterium*, and *Corynebacterium* in VB rinds from both ripening times, and it undergoes a cyclic metabolic process with the biosynthesis of FFAs (Supplementary Material 3).

Acetyl-CoA can undergo a series of reactions that generate butanoic acid, butanal, and butanol, which are compounds with important organoleptic properties in cheese (Figure 5 and Supplementary Material 3). The first step in this metabolic pathway involves the conversion of acetyl-CoA to acetoacetyl-CoA and the transcription of *atoB* transcription (K00626, acetyl-CoA C-acetyltransferase, EC:2.3.1.9). This gene was very highly transcribed overall and mainly associated with *Brevibacterium* at 90 days of ripening (1.7-fold increase), followed by *Corynebacterium* and *Staphylococcus*. Further metabolism of acetoacetyl-CoA involved different bacteria in VB rinds, such as *Brevibacterium, Corynebacterium, Alkalibacterium,* and *Psychrobacter*, and was significantly more transcribed after 90 days of ripening.

The production of organoleptic compounds in cheese is strongly associated with the generation of ATP and NADH. Other enzymes involved in multiple metabolic reactions involving the generation of NADH and organoleptic compounds were also found to be highly transcribed in VB rinds. The aldehyde dehydrogenase (NAD^+^; EC:1.2.1.3), that is coded by ALDH gene (K00128) and was among the 20 most transcribed genes overall and was significantly associated with *Staphylococcus* at day 30 (1.8-fold increase). This enzyme catalyzes the conversion of aldehydes to carboxylates with organoleptic properties, with the generation of NADH. Similarly, the alcohol dehydrogenase (EC:1.1.1.1) can generate NADH with the conversion of alcohols to aldehydes and ketones. The coding gene, *adhP* (K13953), was highly transcribed and significantly associated with *Brevibacterium* and *Corynebacterium* at 90 days of ripening (2.4-fold increase).

Some of the metabolic reactions occurring in cheese rinds might lead to the generation of undesirable compounds. That is the case of the unpleasant odor of skatole (from tryptophan metabolism), and/or of biogenic amines such as, histamine (from histidine), tryptamine (from tryptophan), and tyramine (from tyrosine). The conversion of tryptophan to indole by *tnaA* (K01667, tryptophanase, EC:4.1.99.1) was significantly increased in VB rinds at 90 days of ripening, the overall transcription was low and no transcripts were found that coded for the conversion to skatole (*iad*, K23384, indoleacetate decarboxylase, EC:4.1.1.115). Similarly, no transcripts were found genes involved in the generation of tryptamine and tyramine.

Histidine can be metabolized to histamine by the action of the histidine decarboxylase (EC:4.1.1.22) encoded by *hdc* (K01590). The overall transcription of *hdc* was low but higher in VB rinds at 30 days of ripening (4.7-fold change) and assigned to *Staphylococcus*. The primary-amine oxidase (EC:1.4.3.21) encoded by *tynA* was previously described by our group (Anast et al., 2019) as a putative histamine oxidase in *Brevibacterium* (*Brevibacterium* L261 locus_tag: EB834_15475). This enzyme is hypothesized to be responsible of the first step of the histamine degradation pathway and was significantly transcribed in *Brevibacterium* at 90 days of ripening (11.6-fold change). However, the overall transcription of this gene was low. Other putative proteins described in Anast et al. (2019), including HinD, HinF, HinG, HinH, HinI, and HinL, were screened against the present VB rind dataset and positive hits were considered when the amino acid identity was above 80%. 55 predicted proteins were identified, 51 of which were located in contigs taxonomically assigned as *Brevibacterium*, two as unclassified *Micrococcales* and two as unclassified *Corynebacteriales*. Transcripts were found for all these genes and mostly transcribed at day 90 in *Brevibacterium*, although their overall transcription was low or very low.

## Discussion

Ripening time is one of the key aspects of cheese production, as it greatly influences the organoleptic properties of the products (Smit et al., 2005). During ripening, the microbiota inhabiting the surface of the cheese undergo dynamic changes until it establishes and matures together with the product (Schornsteiner et al., 2014). The investigation of these microbial communities is of great importance and it has been expanded in the last years due to the constant improvement of high-throughput DNA sequencing (HTS) techniques (Quijada et al., 2020a). More precisely, the use of metatranscriptomics, where the entire RNA from a sample is purified, converted to cDNA and subjected to HTS, has gained great popularity in food fermentations, as it allows the investigation of the active microorganisms and metabolic pathways (Pham et al., 2019).

In this study, we performed a metatranscriptomic analysis on VB rinds at two different ripening times. Our group has investigated the VB rind microbiota and the influence of the producing environment, highlighting several taxa to be very abundant in most of the products and producing surfaces regardless the facility or ripening time, such as *Staphylococcus, Brevibacterium, Corynebacterium, Halomonas*, and *Psychrobacter*, among others (Schornsteiner et al., 2014; Quijada et al., 2018, 2020b; Schmitz-Esser et al., 2018; Anast et al., 2019). In this study, VB rind samples were taken at 30 and 90 days of ripening as they represent either young cheeses before they are able to be sold (day 30) or “early ripened” cheeses at the first time that these products are sold (day 90). The ripening of VB can take up to 18 months and this parameter strongly influences the organoleptic properties of the products. VB are usually sold at 3, 6, 10, 12, and 18 months of ripening, with strongly defined textural and organoleptic characteristics.

The ripening time in VB influenced the most active microorganisms and metabolic pathways. *Staphylococcus* was the most active genus at 30 days of ripening, accounting for almost 75% of the transcripts. A shift occurred at 90 days of ripening: *Staphylococcus* was still found to be very active, but other bacteria appeared to be highly active as well, mainly *Brevibacterium, Corynebacterium*. The shift in the transcription pattern is in agreement with our previous studies, as we found *Staphylococcus* (mainly assigned to *Staphylococcus equorum* according to 16S rRNA gene sequences) to be very abundant since the beginning of ripening (when the cheese wheel is extracted from the brine tank and placed in the ripening cellar), throughout the ripening and in the final products. On the other hand, *Brevibacterium* (mainly assigned to *Brevibacterium linens* and *Brevibacterium aurantiacum*) and *Corynebacterium* (mainly assigned to *Corynebacterium variabile*) followed different dynamics, as they appeared at latest ripening times and dominated in the final products (Quijada et al., 2020b). These bacterial groups are not inoculated to the VB during manufacture and were highly abundant in the production environment, that might act as a source of natural inoculation (Quijada et al., 2018).

The production of VB is associated with cold temperatures (approximately 14°C in the ripening cellar) and high salt content, as the cheese wheels are washed with brine regularly. Therefore, most of the active microorganisms encountered here, and also in our previous studies, are able to survive under these conditions. However, the composition of the rind microbes is not stable through time and it is strongly dependent on the availability of nutrients. In the beginning of ripening, lactose is one of the most abundant and important nutrients (McSweeney, 2017). Lactose can be metabolized by different pathways, but we found low activity just in the Leloir pathway at day 30 and mainly associated with *Staphylococcus*. It has been stated that lactose is quickly consumed by LAB at the beginning of ripening (Dugat-Bony et al., 2015) and therefore it is likely to be already exhausted at 30 days of ripening. We did not find transcripts assigned to the LAB bacteria used as starter cultures, in agreement with our previous study where they were only detected when the fresh cheeses were taken out from the brine tank and placed in the ripening cellar (Quijada et al., 2020b). The first steps of lactate metabolism where highly associated with *Staphylococcus* in our study. Similarly, *Staphylococcus* was found to be highly active in the first steps of the degradation of citrate, other of the most important nutrients in milk and fresh cheeses.

Additionally, *Staphylococcus* harbored the greatest number of transcripts at VB rind at day 30 associated with lipases, esterases and proteases, suggesting that *Staphylococcus* is the main driver of the primary events in VB ripening. It was also found to be highly active in the metabolism of some FAA known to be used as energy source after carbohydrate starvation, such as alanine, aspartate and glutamate (Rosenberg and Altemueller, 2001; Monnet et al., 2015; Ganesan and Weimer, 2017). In addition to our studies on VB, *Staphylococcus* is frequently found in other washed, natural and mold rind cheeses (Quigley et al., 2012; Delcenserie et al., 2014; Irlinger et al., 2014; Wolfe et al., 2014), and it is considered as part of the “house” microbiota from washed-rind cheese making plants (Mounier et al., 2006; Bokulich and Mills, 2012). The transcriptomic profiles assigned to *Staphylococcus* in this study enforces its potential role as a “first colonizer” in VB rinds and to exert a pivotal function in VB ripening. According to the gene transcription profiles, the primary metabolic events carried out by *Staphylococcus* in VB rinds at 30 days of ripening set the basis for the secondary events, where the resulting products (mainly FAA and FFA) are metabolized yielding compounds with important organoleptic properties.

At this point*, Brevibacterium* and *Corynebacterium* were found to be the most active bacteria in the secondary metabolic events in VB rinds at day 90. VSC, and concretely methanethiol, which is responsible for the garlic and cooked cabbage aroma and flavor of the products, have gained great attention due to their important flavor and aroma characteristics (Ganesan and Weimer, 2017). We identified the gene encoding the MGL enzyme to be associated with *Brevibacterium* and highly transcribed at day 90. The generation of methanethiol by *Brevibacterium* can lead to the generation of other VSC, such as DMDS and DMTS, that confer garlic and cabbage flavor and aroma to cheeses and have been widely described in Swiss-type cheeses (Weimer et al., 1999; Liu et al., 2004; Marilley and Casey, 2004; McSweeney, 2004; Sourabié et al., 2012). *Brevibacterium* was also highly active in the latest steps of citrate metabolism resulting in the generation of acetoin, diacetyl and 2,3-butanediol. These compounds have been previously associated with buttery, creamy and fruity flavor and aroma in cheeses (McSweeney, 2004, 2017; Escobar-Zepeda et al., 2016).

Both *Brevibacterium* and *Corynebacterium* were found to be very active in the metabolism of branched-chain and aromatic amino acids. The metabolism of phenylalanine leads to the generation of organoleptic compounds such as phenylacetaldehyde and phenylacetate, that has been associated with honey-like, floral, rosy, and violet-aroma of cheeses (McSweeney and Sousa, 2000; Marilley and Casey, 2004; McSweeney, 2004; Escobar-Zepeda et al., 2016). *Brevibacterium* and *Corynebacterium* are considered common members of the washed-rind cheese microbiota and are highly appreciated due to its capacity to confer texture, flavor and aroma to cheeses (McSweeney and Sousa, 2000; Schröder et al., 2011; De Pasquale et al., 2016; Thierry et al., 2017).

Other bacteria such as *Brachybacterium, Alkalibacterium, Yaniella, Halomonas*, and *Psychrobacter* appeared to be active in different metabolic pathways, such as phenylalanine metabolism, FFA biosynthesis or the generation of butanal, mainly at 90 days of ripening. However, the amount of transcripts associated with these taxa was significantly lower than those obtained for *Staphylococcus*, *Brevibacterium* and *Corynebacterium*. These bacteria are also common members of the washed-rind cheeses microbiota (Mounier et al., 2005; Quigley et al., 2012; Dugat-Bony et al., 2016) and were previously found in VB rinds and the producing environment. More precisely, *Halomonas* and *Psychrobacter* were found to be among the most abundant microorganisms in VB rinds and the producing environment (together with *Staphylococcus*, *Brevibacterium*, and *Corynebacterium*; Schornsteiner et al., 2014; Quijada et al., 2018, 2020b). Isolates of *Psychrobacter* were identified in VB rinds and subjected to whole genome sequencing, revealing their important genomic capacities for cheese ripening (Schmitz-Esser et al., 2018). However, we did not find this bacteria to be as active in the investigated cheese metabolic pathways as expected based on their previously detected abundance. The study conducted by Quijada et al. (2018) showed that despite of the high abundance of these genera in the VB producing environment, most of the OTUs found in the rind and the environmental surfaces differed, suggesting that only some representatives of these genera are able to colonize the cheese surface whilst the others inhabit the environmental surfaces favored by the production conditions. In a more recent study (Quijada et al., 2020b), where VB rinds from five different time points and two producers were investigated, it was reported that the presence of *Halomonas* and *Psychrobacter* on the different VB rinds did not follow any specific pattern, suggesting their potential occurrence in VB due to the natural inoculation from the environment. The results obtained here enforce this hypothesis, where *Halomonas* and *Psychrobacter* are naturally occurring in the VB production environment due to their psychro- and halotolerant features and that they spuriously colonize the cheese from the environment, although they do not appear to exert a role on VB ripening as significant as some of the other high abundant microorganisms. Further experiments targeting these bacteria might be conducted to deepen our understanding of their implication in cheese ripening.

In our previous study we identified fungi to be abundant in VB rinds [classified by using ITS amplicon HTS and the UNITE database, (Kõljalg et al., 2005)] although in lower numbers than bacteria according to qPCR measurements. Here, we only identified *ca.* 2% of the transcripts to be assigned as “Fungi” according to the GenBank database. Additional attempts to identify fungal data by using assembly approaches did not improve the amount of classified transcripts.

The advances in shotgun sequencing technologies provide the opportunity to map the microbiome in food industries at an unprecedented depth, highlighting the importance of the resident microbial communities in influencing food quality and safety, as well as the main factors shaping its composition and activities (De Filippis et al., 2021). The use of metatranscriptomics has been recently applied to other cheese models, highlighting the strength of this technique to deepen our understanding of the microbial activities during cheese ripening. Monnet et al. (2016) evaluated the activity of the microorganisms that are added during the manufacture of the surface-ripened reblochon-style cheese, highlighting increase FAA metabolisms of the yeasts (*Debaryomyces hansenii* and *Geotrichum candidum*) at the latest ripening periods investigated (35 days), according to carbohydrate starvation and the generation of ammonia that subsequently increased the pH of the cheese. The knowledge provided by metatranscriptomic analysis has the potential to be applied toward a safer cheese manufacture and/or the enhancement of the quality and organoleptic properties. In the study of De Filippis et al. (2016) they identified that increasing the temperature during the ripening of the Italian Caciocavallo Silano cheese resulted in an increased transcription of genes related with proteolysis, lipolysis and FAA/FFA transport and catabolism in non-starter lactic acid bacteria, and that this increase was correlated with improved aroma profiles and the production of volatile organic compounds. The biochemical and genomic information regarding cheese ripening metabolic pathways available in the literature, as well as the extensive information and models available in up-to-date curated databases, such as the KEGG and the Universal Protein Resource (UniProt), allowed us to draw a metabolic map (as represented in the Figures from the present article) that was used to calculate gene transcription and to infer the potential generation of organoleptic compounds. The information generated here settles the ground for future work involving further sampling in different companies and time points, metabolomic profiles and sensorial characterization of the VB cheeses. This knowledge might be expanded in the future and will be very relevant for cheese makers in order to standardize and accelerate VB ripening while enhancing the organoleptic properties of the product. VB is a regional product from the western part of Austria that has important economic consequences for the population of the region. As described before, VB cheeses are sold from 3 to 18 months, and, although this period influences greatly the properties of the products, it has also high associated costs, in terms of space and maintenance requirements, cleaning, safety and quality controls, brine usage, etc. Therefore, even longer ripening periods can be found, they are rare and most producers do not even produce products with more than 6 months of ripening. The results obtained here can be very important in order to accelerate the production of VB while enhancing the safety and organoleptic properties of the products.

Despite of the strength of metagenomics and metatranscriptomics, there are specific limitations that should be considered. The lack of representative genomes in some of the widely used public databases is one of the main issues. A high fraction of the cheese microbiome is still unknown and might benefit from the use of shotgun sequencing approaches (Walsh et al., 2020). In this study, approximately 15% of the identified CDS did not retrieve any taxonomic assignment against the GenBank database and more than 50% of the CDS did not get any assigned function against KEGG. Additional culturing and functional approaches might be conducted in order to overcome this issue, as the amount of sequencing data that is generated nowadays exceeds the number of molecular or culturing-based data. *Staphylococcus, Brevibacterium*, and *Corynebacterium*, were found to be the most active microorganisms overall, which enforces their key role for the proper manufacture and development of the organoleptic properties of VB. Further studies might target these bacteria by both culture-dependent and independent approaches, in order to deepen their potential use as ripening cultures to be used in VB to standardize and accelerate the ripening of this product.

## Data Availability Statement

The datasets presented in this study can be found in online repositories. The names of the repository/repositories and accession number(s) can be found at: https://www.ebi.ac.uk/ena, PRJEB48589.

## Author Contributions

MD, ES, and SS-E devised the study design. MD collected the samples and performed the nucleic acids purifications. NMQ drafted the manuscript and performed the bioinformatic analysis and the data visualization. ES, MD, MW, NMQ, and SS-E evaluated and discussed the results and reviewed, edited, and prepared the submitted version of the manuscript. ES and MW acquired the funding for the study. All authors contributed to the article and approved the submitted version.

## Funding

The competence centre FFoQSI is funded by the Austrian ministries BMVIT, BMDW and the Austrian provinces Niederösterreich, Upper Austria and Vienna within the scope of COMET—Competence Centers for Excellent Technologies. The programme COMET is handled by the Austrian Research Promotion Agency FFG. This study was also supported and funded with the MASTER project, which has received funding from the European Union’s Horizon 2020 Research and Innovation Programme under grant agreement No 818368. We gratefully acknowledge funding for high-throughput sequencing by the provincial government of Vorarlberg, No IIb-13.06-12/2018-2 8; VetMed Proj FA21018040.

## Conflict of Interest

NMQ and MW were employed by the company FFoQSI GmbH.

The remaining authors declare that the research was conducted in the absence of any commercial or financial relationships that could be construed as a potential conflict of interest.

## Publisher’s Note

All claims expressed in this article are solely those of the authors and do not necessarily represent those of their affiliated organizations, or those of the publisher, the editors and the reviewers. Any product that may be evaluated in this article, or claim that may be made by its manufacturer, is not guaranteed or endorsed by the publisher.

## Footnotes

^1^ https://www.bioinformatics.babraham.ac.uk/projects/fastqc/

^2^ https://inkscape.org
