# Supplementary material for: Metatranscriptomic Analyses Unravel Dynamic Changes in the Microbial and Metabolic Transcriptional Profiles in Artisanal Austrian Hard-Cheeses During Ripening
Source: Front Microbiol. 2022 Mar 1;13:813480. doi: 10.3389/fmicb.2022.813480 (PMC8921697; doi:10.3389/fmicb.2022.813480)
Supplement: Supplementary file 1 [file Data_Sheet_1.pdf]

Supplementary File 2

Methionine can be metabolized to *S*-adenosylmethionine (SAM), which is vital for one-carbon transfer reactions within the cell. The *metK* gene (K00789) that codes for the methionine adenosyltransferase (EC:2.5.1.6) involved in this conversion was highly transcribed in VB rinds but did not show significant differences between 30 or 90 days of ripening. However, its transcription was mainly associated with *Staphylococcus* in VB from day 30 and with *Brevibacterium, Corynebacterium,* and *Staphylococcus* at day 90. SAM can be converted to L-homocysteine by different pathways, such as from *S*-adenosyl-L-homocysteine, controlled by the transcription of *ahcY* (K01251, adenosylhomocysteinase, EC:3.3.1.1), or from *S*-D-ribosyl-L-homocysteine, controlled by *luxS* (K07173, *S*-ribosylhomocysteine lyase, EC:4.4.1.21). These two genes were found to be highly transcribed in VB overall. However, there were significant differences in their transcription according to the ripening time and the microbiota involved. *achY* was significantly more transcribed in *Corynebacterium* and *Brevibacterium* in VB rinds from day 90, whereas *luxS* was significantly more transcribed in *Staphylococcus* from day 30.

Cysteine and methionine are connected through homocysteine and cystathionine. Homocysteine can be methylated to yield Methionine by the action of different methyltransferases, such as the homocysteine *S*-methyltransferase (EC:2.1.1.10) encoded by *metE* (K00549). As it can be seen in Figure 3, *metE* was highly transcribed in VB rinds overall and significantly more transcribed at 30 days of ripening and associated with *Staphylococcus* (2.5-fold change). Homocysteine can be generated from cystathionine with the generation of pyruvate and ammonia by the action of the CBL coded by *metC* and *patB*, as described before. Alternatively, homocysteine can be converted to cystathionine by the action O-acetylserine dependent cystathionine beta-synthase (EC:2.5.1.134), encoded by *mccA* (K17216). *mccA* transcription was associated with *Staphylococcus* at both ripening times but was significantly higher in day 30 (27.7-fold change). Additionally, homocysteine can incorporate serine into the cycle by the cystathionine beta-synthase (EC:4.2.1.22), generating cystathionine. The *cbs* gene (K01697) coding for this enzyme was significantly more transcribed in VB rinds from day 90, and was associated with *Brevibacterium* and unclassified *Micrococcaceae*, although its overall transcription was low. Other amino acids, such as aspartate and threonine, can also be incorporated to the cycle via L-homoserine. L-homoserine can be metabolized to O-succinyl-L-homoserine or to O-acetyl-L-homoserine by the action of homoserine O-acetyltransferase/O-succinyltransferase (EC:2.3.1.31, EC:2.3.1.46) encoded by *metX* (K00641). This gene showed a medium transcription overall and was significantly more transcribed in *Staphylococcus* at day 30 (3.2-fold change compared to day 90). These two products can be metabolized to cystathionine by the action of cystathionine gamma-synthase (EC:2.5.1.48), encoded by the *metB* gene (K01739), which was significantly more transcribed in *Brevibacterium, Corynebacterium*, and unclassified *Micrococcaceae* from VB rinds ripened for 90 days. O-acetyl-L-homoserine can also be converted to homocysteine by the action of the same cystathionine gamma-

synthase and by O-acetylhomoserine aminocarboxypropyltransferase (EC:2.5.1.49). The latter enzyme is coded by *metY* (K01740), which was highly transcribed overall and was significantly more transcribed in *Staphylococcus* in VB rinds with 30 days of ripening (6.4-fold change).

Finally, cysteine can be generated from cystathionine, generating α-ketobutyrate and ammonia, by the action of cystathionine gamma-lyase (CGL, EC:4.4.1.1), which is coded by *mccB* (K17217) as described before. Further metabolism of cysteine can generate pyruvate by the action of different enzymes. *sseA* (K01011) codes for this reaction, was highly transcribed overall and significantly more transcribed in VB rinds from day 90, associated mainly with *Brevibacterium* and *Corynebacterium*.

Despite of methanethiol being the most important VSC for cheese organoleptic properties, methionine can be the source for the generation of other organoleptic compounds. A transamination reaction catalyzed by an aminotransferase can lead to the conversion of methionine and α-ketoglutarate into glutamate and the sulfur-containing keto acid 4-methylthio-2-oxobutanoate (KMBA). KMBA can be subsequently converted to the organoleptic compounds methanethiol and methional by either chemical decomposition or enzymatic conversion due to a demethiolating activity. However, the genes involved in these reactions, *mtnE/mtnV* (K08969, L-glutamine---4-(methylsulfanyl)-2-oxobutanoate aminotransferase, EC:2.6.1.117), and *tyrB* (K00832, aromatic-amino-acid transaminase, EC:2.6.1.57) had a low transcription overall (transcription was associated with *Staphylococcus, Halomonas* and *Psychrobacter*). KMBA can also yield methanethiol via the formation of 3-methylthiopropionic acid, but the intermediates are yet to be identified.
