# Supplementary material for: Metatranscriptomic Analyses Unravel Dynamic Changes in the Microbial and Metabolic Transcriptional Profiles in Artisanal Austrian Hard-Cheeses During Ripening
Source: Front Microbiol. 2022 Mar 1;13:813480. doi: 10.3389/fmicb.2022.813480 (PMC8921697; doi:10.3389/fmicb.2022.813480)
Supplement: Supplementary file 2 [file Data_Sheet_2.pdf]

Supplementary File 3

The catabolism of branched-chain amino acids (leucine, isoleucine and valine) involves a series of reactions that generate different branched-chain fatty acids with important organoleptic properties. The first steps in the degradation of these amino acids are similar between them and involves the action of amino transferases, dehydrogenases, and acyl transferases that generate acyl-enoyl-CoA compounds as intermediate products (2-methylprop-2-enoyl-CoA from valine, 2-methylbut-2-enoyl-CoA from isoleucine and 3-methylbut-2-enoyl-CoA from leucine). Most of the genes coding for the enzymes involved in these conversions were significantly more transcribed at day 30 of ripening in *Staphylococcus* (Figure 5). Additionally, some of them were among the most transcribed genes in the entire study overall. That was the case of *pdhD* (K00382, dihydrolipoamide dehydrogenase, EC:1.8.1.4), *bkdA2* (K00167, 2-oxoisovalerate dehydrogenase E1 component beta subunit, EC:1.2.4.4) and *bkdB* (K09699, 2-oxoisovalerate dehydrogenase E2 component (dihydrolipoyl transacylase), EC:2.3.1.168). *pdhD* is involved in multiple other reactions, such as the conversion of pyruvate to acetyl-CoA, and was among the 30 most transcribed genes in the entire study. The intermediate acyl-enoyl-CoA compounds could be converted to their corresponding branched chain FFAs by the action of phosphatases and acyl kinases, with the concomitant generation of ATP. Similarly, due to the activity of the branched-chain amino acid transaminase (EC:2.6.1.42), leucine, isoleucine and valine can be converted into their respective carboxylic acids (4-methyl-2-oxopentanoate, (S)-3-methyl-2-oxopentanoate, and 3-methyl-2-oxobutanoate, respectively), which also harbor important organoleptic properties. This enzyme is encoded by *ilvE* (K00826) that was highly transcribed at both ripening times, but the differences were not significant between the ripening times. Interestingly, the transcription of *ilvE* was mainly associated with *Staphylococcus* at day 30 and with *Brevibacterium* at day 90.

The next steps in the metabolism of leucine differs from those of valine and isoleucine and so they were the final compounds, as leucine metabolism yields to the formation of acetyl-CoA whereas propionyl-CoA is formed from valine and isoleucine (Figure 5). Strikingly, most of the genes involved in these latest reactions of valine, isoleucine and leucine degradation were significantly more transcribed at day 90 and mainly associated with *Brevibacterium* and *Corynebacterium*. However, the overall transcription of these genes was not high, except for *paaF* (K01692, enoyl-CoA hydratase, EC:4.2.1.17) and *fadA* (K00632, acetyl-CoA acyltransferase, EC:2.3.1.16). *fadA* is involved in a wide number of reactions and it was among the 100 most transcribed genes overall.

Propionyl-CoA is one of the final products after isoleucine and valine metabolism, but can also be generated after the metabolism of threonine and methionine (Figure 5). The generation of propionyl-CoA from threonine and methionine requires their previous conversion into α-ketobutyrate (in the case of methionine, with the concomitant generation of methanethiol, as described before). The conversion of α-ketobutyrate to propionyl-CoA is controlled by *pflD* (K00656, formate *C*-acetyltransferase, EC:2.3.1.54),

which was significantly more transcribed at day 90 in *Brachybacterium* and *Alkalibacterium*. Propionyl-CoA can be further metabolized to generate propionate that confers organoleptic properties for the cheese, with the concomitant generation of ATP. The genes *pta* (K00625, phosphate acetyltransferase, EC:2.3.1.8) and *ackA* (K00925, acetate kinase, EC:2.7.2.1) are involved in these reactions and were highly transcribed in VB at both ripening and mainly associated with *Staphylococcus* at day 30 and to *Staphylococcus* and *Corynebacterium* at day 90.

Propionyl-CoA can also be generated from pyruvate after the tricarboxylic acids (TCA) cycle via R-methylmalonyl (Figure 5). Most of the genes in this pathway were very highly transcribed in VB rinds. That was the case of *mqo* (K00116, malate dehydrogenase (quinone), EC:1.1.5.4), that was the fifth more transcribed gene overall, and mainly associated with *Staphylococcus* at both ripening times. Pyruvate has a key role in cell metabolism and can be generated from lactose, lactate, and citrate (as described in the previous section), as well as from different FAA, including alanine, cysteine, glutamate, and serine (Figure 5).

Alanine is produced and metabolized during carbohydrate starvation and cheese aging. Alanine can be transaminated or deaminated to yield pyruvate. Several genes are involved in this reaction, such as *AGXT* (K00830, alanine-glyoxylate transaminase, EC:2.6.1.44), which was highly transcribed and significantly more transcribed at day 30 in *Staphylococcus*. Alanine can be produced from aspartate by a decarboxylation reaction, although the overall transcription of this pathway was low. Alternatively, aspartate can be deaminated to produce oxaloacetate, ammonia, and hydrogen peroxide. This reaction is controlled by *nadB* (K00278, L-aspartate oxidase, EC:1.4.3.16) which was significantly more transcribed in *Staphylococcus* at day 30 (7.0-fold change). Additionally, the aspartate aminotransferase (EC:2.6.1.1) coded by *aspB* (K00812) is involved in the conversion of aspartate to oxaloacetate. The same enzyme is also involved in the conversion of phenylalanine to phenylpyruvate described before (Figure 4). *aspB* was significantly more transcribed in *Brevibacterium, Brachybacterium, Leucobacter,* and *Corynebacterium* from VB rinds ripened for 90 days, although the overall transcription of this gene was medium.

Glutamate is one of the most abundant amino acids in milk caseins and FAAs in cheese. Glutamate can be degraded to α-ketoglutarate with the generation of NAD(P)H and ammonia by the action of dehydrogenases and transaminases. *gudB* (K00260, glutamate dehydrogenase, EC:1.4.1.2) was very highly transcribed overall and significantly associated with *Staphylococcus* at 30 days of ripening (Figure 5). The generated α-ketoglutarate can enter the TCA cycle or be used as a substrate for transamination reactions and favors the catabolism of other FAAs, as they work as amino group acceptors.

In the previous section it was described that serine could enter the metabolic cycle of the *S*-containing amino acids by the action of *cbs*, although the overall transcription of this gene was low in VB rinds. Serine can also be converted to pyruvate by the action of L-

serine dehydratase (EC:4.3.1.17) which is coded by *sdaA/sdaB* (K01752). This gene was highly transcribed and significantly more transcribed at day 30 in *Staphylococcus* (3.3-fold change). Ser can be formed from glycine, regulated by the highly transcribed *glyA* (K00600, glycine hydroxymethyltransferase, EC:2.1.2.1). *glyA* was significantly more transcribed at day 30 in *Staphylococcus*. *Brevibacterium* and *Corynebacterium* were the main responsible for this conversion in VB ripened for 90 days. Gly can be formed from Threonine with the concomitant generation of acetaldehyde. This reaction was conducted mainly by *Brevibacterium* at both ripening times, although the *ltaE* (K01620, threonine aldolase, EC:4.1.2.48) gene involved did not show a high transcription overall.

As described before, FFA may originate from the degradation of milk fats by the action of lipases and esterases. Additionally, they can also be formed after the breakdown of FAA or synthesized *ex-novo* from the pyruvate obtained after FAA catabolism (Figure 5). Pyruvate can be converted to acetyl-CoA by different reactions (as also described in the *Metabolism of residual lactose, lactate and citrate* section, Figure 2). Acetyl-CoA receives the acyl carrier protein (acp) from Malonyl-[acp]. Malyonyl-[acp] is also produced from acetyl-CoA involving a series of genes that were mainly associated with *Staphylococcus, Brevibacterium*, and *Corynebacterium* in VB rinds from both ripening times, and it undergoes a cyclic metabolic process with the biosynthesis of FFAs. This cyclic process adds two carbon atoms per cycle. Propionyl-CoA can substitute malonyl-CoA, leading to an odd-numbered FFA chain. Different bacteria were found to be active in this series of reactions, such as *Staphylococcus, Brevibacterium, Corynebacterium, Halomonas, Yaniella, Marinobacter,* and *Alkalibacterium*. Different genes are involved in this metabolic pathway, and the gene that was most highly transcribed overall was *fabG* (K00059), which codes for 3-oxoacyl-[acyl-carrier protein] reductase (EC:1.1.1.100). This gene was among the 100 most transcribed genes overall and did not show significant differences in transcription between VB rinds at 30 or 90 days of ripening. It was mainly associated with *Staphylococcus* after 30 days of ripening, followed by *Brevibacterium* and *Psychrobacter*. At 90 days of ripening, *fabG* activity was mainly associated with *Brevibacterium,* followed by *Staphylococcus, Psychrobacter* and *Corynebacterium*. The genes *fabZ* (K02372) and *fabF* (K09458) were also highly transcribed in both ripening times, where the transcription in *Staphylococcus* was significantly increased at 30 days of ripening. At certain points of the cycle, the medium-chain acyl-[acyl-carrier-protein] hydrolase (EC:3.1.2.21) can release FFA from their corresponding acyl-[acp]. This enzyme is coded by *mch* (K01071), which did not show a high transcription profile overall nor significant differences in its transcription in VB between ripening times. Its transcription was associated with *Alkalibacterium, Halomonas,* unclassified *Actinobacteria* and unclassified *Corynebacteriales*.

In addition to this pathway and to those described in the *Metabolism of residual lactose, lactate and citrate* section, acetyl-CoA can be involved in the formation of butanoic acid, butanal, and butanol, which are compounds with important organoleptic properties in cheese. This metabolic pathway involves a series of reactions that starts with the conversion of acetyl-CoA to acetoacetyl-CoA controlled by *atoB* transcription (K00626,

acetyl-CoA C-acetyltransferase, EC:2.3.1.9). *atoB* was very highly transcribed overall and mainly associated with *Brevibacterium* at 90 days of ripening (1.7-fold change), followed by *Corynebacterium* and *Staphylococcus*. As it can be seen in Figure 5, most of the genes coding the enzymes involved in different reactions after acetotacetyl-CoA formation were significantly more transcribed in VB rinds at day 90, and the main bacteria involved were *Brevibacterium, Corynebacterium, Alkalibacterium,* and *Psychrobacter*. One of the intermediates of this pathway, crotonyl-CoA, can also be generated from Glu after its conversion into α-ketoglutarate. The genes involved in the conversion of α-ketoglutarate to crotonyl-CoA, *L2HGDH* (K00109, L-2-hydroxyglutarate dehydrogenase, EC:1.1.99.2), *gctA* (K01039, glutaconate CoA-transferase, EC:2.8.3.12) and *gctB* (K01040, glutaconate CoA-transferase, EC:2.8.3.12), were significantly more transcribed in *Brevibacterium* at 90 days of ripening, although the transcription of this pathway was low overall.

The production of organoleptic compounds in cheese is strongly associated with the generation of ATP and NADH. Other enzymes involved in multiple metabolic reactions involving the generation of NADH and organoleptic compounds were also found to be highly transcribed in VB rinds. The aldehyde dehydrogenase (NAD+) (EC:1.2.1.3), that is coded by *ALDH* (K00128) and was among the 20 most transcribed genes overall and was significantly associated with *Staphylococcus* at day 30 (1.8-fold change). This enzyme catalyses the conversion of aldehydes to carboxylates with organoleptic properties, with the generation of NADH. Similarly, the alcohol dehydrogenase (EC:1.1.1.1) can generate NADH with the conversion of alcohols to aldehydes and ketones. The coding gene, *adhP* (K13953), was highly transcribed and significantly associated with *Brevibacterium* and *Corynebacterium* at 90 days of ripening (2.4-fold change).

Some of the metabolic reactions occurring in cheese rinds might lead to the generation of undesirable compounds. That is the case of the unpleasant odor of skatole (from tryptophan metabolism), and/or of biogenic amines such as, histamine (from histidine), tryptamine (from tryptophan) and tyramine (from tyrosine). The conversion of tryptophan to indole by *tnaA* (K01667, tryptophanase, EC:4.1.99.1) was significantly increased in VB rinds at 90 days of ripening, the overall transcription was low and no transcripts were found that coded for the conversion to skatole (*iad*, K23384, indoleacetate decarboxylase, EC:4.1.1.115). Similarly, no transcripts were found genes involved in the generation of tryptamine and tyramine (Supplementary File 1).

Histidine can be metabolized to histamine by the action of the histidine decarboxylase (EC:4.1.1.22) encoded by *hdc* (K01590). The overall transcription of *hdc* was low but higher in VB rinds at 30 days of ripening (4.7-fold change) and assigned to *Staphylococcus*. The primary-amine oxidase (EC:1.4.3.21) encoded by *tynA* was previously described by our group (Anast et al., 2019) as a putative histamine oxidase in *Brevibacterium* (*Brevibacterium* L261 locus_tag: EB834_15475). This enzyme is hypothesized to be responsible of the first step of the histamine degradation pathway and

was significantly transcribed in *Brevibacterium* at 90 days of ripening (11.6-fold change). However, the overall transcription of these gene was low. Other putative proteins described in Anast et al. (2019), including HinD, HinF, HinG, HinH, HinI and HinL, were screened against the present VB rind dataset and positive hits were considered when the amino acid identity was above 80%. 55 predicted proteins were identified, 51 of which were located in contigs taxonomically assigned as *Brevibacterium*, 2 as unclassified *Micrococcales* and 2 as unclassified *Corynebacteriales*. Transcripts were found for all these genes and mostly transcribed at day 90 in *Brevibacterium*, although their overall transcription was low or very low.
